# Two new species of *Oberwinklerozyma* and *Rosettozyma (Microbotryomycetes)* from Yunnan, southwestern China

**DOI:** 10.3897/mycokeys.141.204237

**Published:** 2026-10-05

**Authors:** Long Tian, Chun-Yue Chai, Qiu-Hong Niu, Feng-Li Hui

**Affiliations:** 1 School of Life Science, Nanyang Normal University, Nanyang 473061, China School of Life Science, Nanyang Normal University Nanyang China; 2 State Key Laboratory of Motor Vehicle Biofuel Technology, Nanyang 473000, China State Key Laboratory of Motor Vehicle Biofuel Technology Nanyang China

**Keywords:** Basidiomycetous yeast, phylogenetic analysis, rotting wood, taxonomy

## Abstract

*Oberwinklerozyma* and *Rosettozyma* comprise a relatively small number of described species worldwide. Although several novel species from China have recently been reported, the species diversity of these two genera remains incompletely understood. In this study, two novel yeast species, *Oberwinklerozyma jinghongensis***sp. nov**. and *Rosettozyma fusiformis***sp. nov**., are proposed to accommodate three strains isolated from rotting wood collected in Xishuangbanna Primeval Forest Park, Jinghong, Yunnan Province, southwestern China. Detailed descriptions and illustrations are provided. Genus-level phylogenetic relationships were inferred based on sequence analyses of the D1/D2 domain of the large subunit (LSU) rRNA gene and the internal transcribed spacer (ITS) region. This study expands current knowledge of the diversity and taxonomy of *Oberwinklerozyma* and *Rosettozyma*.

## Introduction

The genus *Oberwinklerozyma* was first proposed by Wang et al. (2015a) to accommodate three anamorphic species, *O. silvestris*, *O. straminea*, and *O. yarrowii*, which had previously been recognized as members of the *yarrowii* clade within the *Microbotryomycetes* (Wang et al. 2015b), a class in which many phylogenetically related members are mycoparasites (Schoutteten et al. 2023). Subsequently, *O. nepetae* and *O. dicranopteridis*, isolated from leaves of *Nepeta* sp. and *Dicranopteris dichotoma* in China, respectively, were described as two additional species of *Oberwinklerozyma* (Li et al. 2020). Based on multigene phylogenetic analyses, this genus is closely related to the genera *Pseudohyphozyma* and *Slooffia* (Wang et al. 2015a). However, species of *Oberwinklerozyma* are characterized by Q-9 as the major coenzyme Q system and by their ability to assimilate raffinose and *myo*-inositol, whereas members of *Pseudohyphozyma* and *Slooffia* possess Q-10 and are unable to utilize these two carbon sources (Wang et al. 2015a). Species of *Oberwinklerozyma* have frequently been isolated from different parts of woody plants and, less commonly, from decaying mushrooms (Fonseca and van Uden 1991; Golubev and Scorzetti 2010; Li et al. 2020).

The genus *Rosettozyma* was recently established by Li et al. (2020) to accommodate three novel species, namely *R. petaloides*, *R. cystopteridis*, and *R. motuoensis*. More recently, the phylloplane yeast *R. turpiniae*, isolated from China, was added to the genus (Sun et al. 2025). Phylogenetically, *Rosettozyma* is placed in the family *Rosettozymaceae*, order *Rosettozymales*, near the base of the *Microbotryomycetes* (Li et al. 2020). Phenotypically, species of *Rosettozyma* produce whitish to cream-colored, butyrous colonies and reproduce by polar budding, often forming rosette-like clusters (Li et al. 2020; Sun et al. 2025). All currently known members of *Rosettozyma* are nonfermentative and are capable of assimilating a variety of carbon sources, but they do not assimilate *myo*-inositol or methanol (Li et al. 2020; Sun et al. 2025). Species of *Rosettozyma* are frequently isolated as epiphytes from the leaves of various plants and are regarded as important phyllosphere-inhabiting yeasts (Li et al. 2020; Sun et al. 2025).

Over the past few years, several novel species of *Oberwinklerozyma* and *Rosettozyma* have been described (Li et al. 2020; Sun et al. 2025). Nevertheless, the species diversity within these two genera remains only partially documented. In this study, two novel yeast species, *Oberwinklerozyma jinghongensis* sp. nov. and *Rosettozyma fusiformis* sp. nov., isolated from rotting wood collected in the Xishuangbanna Tropical Rainforest, Yunnan Province, China are described. Detailed morphological descriptions and phylogenetic analyses are provided to support their taxonomic placement.

## Materials and methods

### Sample collection and yeast isolation

Rotting wood samples were collected from Xishuangbanna Primeval Forest Park, Jinghong, Yunnan Province, China (21°98'N, 100°88'E). The study area is located south of the Tropic of Cancer and has a tropical monsoon climate characterized by ample sunlight, abundant rainfall, and high biodiversity. The average annual temperature is approximately 21 °C, and the mean annual precipitation ranges from 1,200 to 1,700 mm. The region is frost- and snow-free throughout the year, with 108–146 foggy days annually. Notably, ant activity was observed in the rotting wood at the time of sampling. Each sample was placed in a sterile plastic bag, kept on ice, and transported to the laboratory within 24 h for subsequent processing. Yeast strains were isolated from the rotting wood samples following the methods described by Zheng et al. (2017). Briefly, each sample (1 g) was added to 20 mL of sterile yeast extract–malt extract (YM) broth containing 1% glucose, 0.5% peptone, 0.3% yeast extract, and 0.3% malt extract, pH 5.4, supplemented with 0.02% chloramphenicol in a 150 mL Erlenmeyer flask. The cultures were incubated at 25 °C for 3 days on a rotary shaker. Subsequently, 0.1 mL of the enrichment culture and appropriate decimal dilutions were spread onto YM agar plates supplemented with 0.02% chloramphenicol and incubated at 25 °C for 3–4 days. Colonies showing distinct yeast-like morphologies were selected and purified by repeated streaking on YM agar. Purified strains were preserved in YM broth containing 20% (v/v) glycerol at –80 °C for further study.

### Phenotypic characterization

Morphological, physiological, and biochemical characteristics were examined according to standard methods used in yeast taxonomy (Kurtzman et al. 2011). Colony morphology was observed after incubation on YM agar at 25 °C for 7 days. Cellular morphology was examined in YM broth after incubation at 25 °C for 3 days using a Leica DM2500 light microscope (Leica, Wetzlar, Germany). Mycelium formation was investigated by slide culture on corn meal agar (CMA; 2% cornmeal infusion and 2% agar) at 25 °C for 2 weeks. The ability to produce ballistoconidia was tested using the inverted-plate method (do Carmo-Sousa and Phaff 1962) on CMA at 15 and 25 °C for 2 weeks, and discharged spores were examined microscopically after 3–14 days. To investigate potential sexual reproduction, individual strains and pairwise combinations of strains were inoculated onto CMA, potato dextrose agar (PDA; 20% potato infusion, 2% glucose, and 2% agar), and V8 agar (10% V8 juice and 2% agar) and incubated at 15 and 25 °C for up to 6 weeks, with observations made at 2-week intervals (Li et al. 2020; Sun et al. 2025). Carbon and nitrogen source assimilation tests were performed in liquid media, and starved inocula were used for nitrogen assimilation tests. Glucose fermentation was assessed using Durham fermentation tubes. Growth at different temperatures was evaluated on YM agar at temperatures ranging from 15 to 37 °C. The newly proposed taxa were registered in the MycoBank database (https://www.mycobank.org; accessed 16 June 2026).

### DNA extraction, PCR amplification, sequencing

Genomic DNA was extracted from actively growing yeast cells using the Ezup Column Yeast Genomic DNA Purification Kit (Sangon Biotech, Shanghai, China) according to the manufacturer’s instructions. The D1/D2 domain of the large subunit (LSU) rRNA gene and the internal transcribed spacer (ITS) region were amplified using the primer pairs NL1/NL4 (Kurtzman and Robnett 1998) and ITS1/ITS4 (White et al. 1990), respectively. PCR amplifications were performed in 25 μL reaction mixtures containing 9.5 μL of nuclease-free water, 12.5 μL of 2× Taq PCR Master Mix with blue dye (Sangon Biotech, Shanghai, China), 1 μL of genomic DNA template, and 1 μL of each primer. Amplification was carried out using an AB 2720 thermal cycler (Applied Biosystems, Foster City, CA, USA) under the following conditions: initial denaturation at 98 °C for 2 min; 35 cycles of denaturation at 98 °C for 10 s, annealing at 52 °C for 10 s, and extension at 72 °C for 15 s; followed by a final extension at 72 °C for 5 min (Chai et al. 2023). PCR products were purified using a SanPrep Column PCR Product Purification Kit (Sangon Biotech, Shanghai, China) and sequenced in both directions by Sangon Biotech Co., Ltd. (Shanghai, China). Forward and reverse sequences were assembled and edited using BioEdit v. 7.1.3.0 (Hall 1999). All newly generated sequences were deposited in GenBank (https://www.ncbi.nlm.nih.gov/genbank/).

### Phylogenetic analyses

Sequences generated in this study were compared with reference sequences retrieved from GenBank using BLASTn searches (Table 1). Reference taxa were selected based on BLAST results and recent phylogenetic studies (Wang et al. 2015a, 2015b; Li et al. 2020; Schoutteten et al. 2023; Sun et al. 2025). Sequences for each locus were aligned separately using MAFFT v. 7 with the L-INS-i algorithm (Katoh and Standley 2013), and the alignments were manually adjusted in BioEdit v. 7.1.3.0 where necessary. Individual alignments were concatenated using PhyloSuite v. 1.2.3 (Zhang et al. 2020).

**Table 1.** Sequences used in phylogenetic analysis. Entries in bold are newly generated in this study.

**Species**	**Strain no**.	**Country**	**GenBank accession no**.	
			**D1/D2**	**ITS**
*Colacogloea cycloclastica*	CBS 8448^T^	Thailand	NR_154750	NG_058729
*Colacogloea philyla*	CBS 6272^T^	South Africa	NR_073274	NR_073274
*Colacogloea retinophila*	CBS 8446 ^T^	South Africa	NR_154830	NG_058994
*Colacogloea terpenoidalis*	CBS 8445^T^	Malaysia	NR_154749	NG_058995
*Erythrobasidium elongatum*	CBS 8080^T^	Australia	NG_059254	NR_073306
*Erythrobasidium hasegawianum*	JCM 1545^T^	Japan	AF131058	NR_111008
*Heterogastridium pycnidioideum*	ATCC MYA-4631^T^	UK	GU291290	GU291276
*Meredithblackwellia eburnea*	CBS 12589^T^	Guyana	NR_120214	NG_058333
*Oberwinklerozyma dicranopteridis*	CGMCC 2.3441^T^	China	MK050426	NR_174791
*Oberwinklerozyma nepetae*	CGMCC 2.5824^T^	China	MK050427	MK050427
***Oberwinklerozyma jinghongensis***	**NYNU 181056^T^**	**China**	**MK682806**	**MK682798**
***Oberwinklerozyma jinghongensis***	**NYNU 181073**	**China**	**PZ499776**	**PZ499777**
*Oberwinklerozyma silvestris*	CBS 11420^T^	Russia	NR_163533	GQ121044
*Oberwinklerozyma straminea*	CBS 10976^T^	Russia	NR_155583	EU872489
*Oberwinklerozyma yarrowii*	CBS 7417^T^	Portugal	NR_073328	NG_058357
*Pseudohyphozyma bogoriensis*	CBS 4101^T^	Indonesia	NG_058376	NR_073291
*Rosettozyma cystopteridis*	CGMCC 2.2619^T^	China	MK050399	NR_174038
***Rosettozyma fusiformis***	**NYNU 181054^T^**	**China**	**MK682808**	**MK682801**
*Rosettozyma motuoensis*	CGMCC 2.5819^T^	China	MK050400	NR_174039
*Rosettozyma petaloides*	CGMCC 2.3446^T^	China	MK050395	NR_174037
*Rosettozyma turpiniae*	CGMCC 2.6626^T^	China	OL853573	OL853573
*Rosettozyma* sp.	BRT 367	Brazil	OR430102	–
*Rosettozyma* sp.	BRT 395	Brazil	OR430103	–
*Slooffia cresolica*	CBS 7998^T^	Netherlands	NG_057666	NR_073313
*Slooffia tsugae*	CBS 5038^T^	–	NG_057672	NR_155840
*Spencerozyma crocea*	CBS 2029^T^	USA	NG_057723	NR_103609
*Spencerozyma siamensis*	DMKU13-2^T^	Thailand	LC195008	LC315095
*Trigonosporomyces hylophilus*	CBS 6226^T^	South Africa	NG_058423	NR_154536
*Vonarxula javanica*	CBS 5236^T^	Jawa	NG_057667	NR_111079
*Phyllozyma dimennae*	JCM 8762^T^	New Zealand	AB644404	NR_144764
*Phyllozyma novozealandica*	CBS 7895^T^	New Zealand	NG_058373	NR_144765

Note. Strains marked with ^“T”^ are ex-type.

Phylogenetic analysis was conducted using the maximum likelihood (ML) method based on the concatenated D1/D2 and ITS sequences. ML analysis was performed in MEGA v. 7 (Kumar et al. 2016) under the GTR+I+G substitution model, with branch support assessed using 1,000 bootstrap replicates (Felsenstein 1985). Bootstrap values (BS) ≥50% were considered significant.

## Results

### Yeast isolation and diversity

During a survey of yeasts associated with rotting wood in the Xishuangbanna Tropical Rainforest, yeasts were recovered from over 90% of the samples examined, yielding 96 strains from 52 samples. Based on D1/D2 and ITS sequence analyses, the majority of the isolates were assigned to known species, including *Apiotrichum laibachii*, *Candida tropicalis*, *Cutaneotrichosporon cutaneum*, *Hemisphaericaspora subhashii*, *Kurtzmaniella natalensis*, *Lachancea thermotolerans*, *Ogataea siamensis*, *Petasospora bimundalis*, *Saitozyma podzolica*, *Saturnispora diversa*, *Schwanniomyces vanrijiae*, *Scheffersomyces insectosa*, *Scheffersomyces stipitis*, *Sugiyamaella americana*, *Sugiyamaella valdiviana*, *Torulaspora delbrueckii*, *Wickerhamomyces pijperi*, *Wickerhamiella spandovensis*, and *Yamadazyma akitaensis*. However, three isolates could not be assigned to any known species and are therefore considered to represent two potential novel species requiring further characterization.

### Phylogeny of novel yeast species

**Figure 1.**
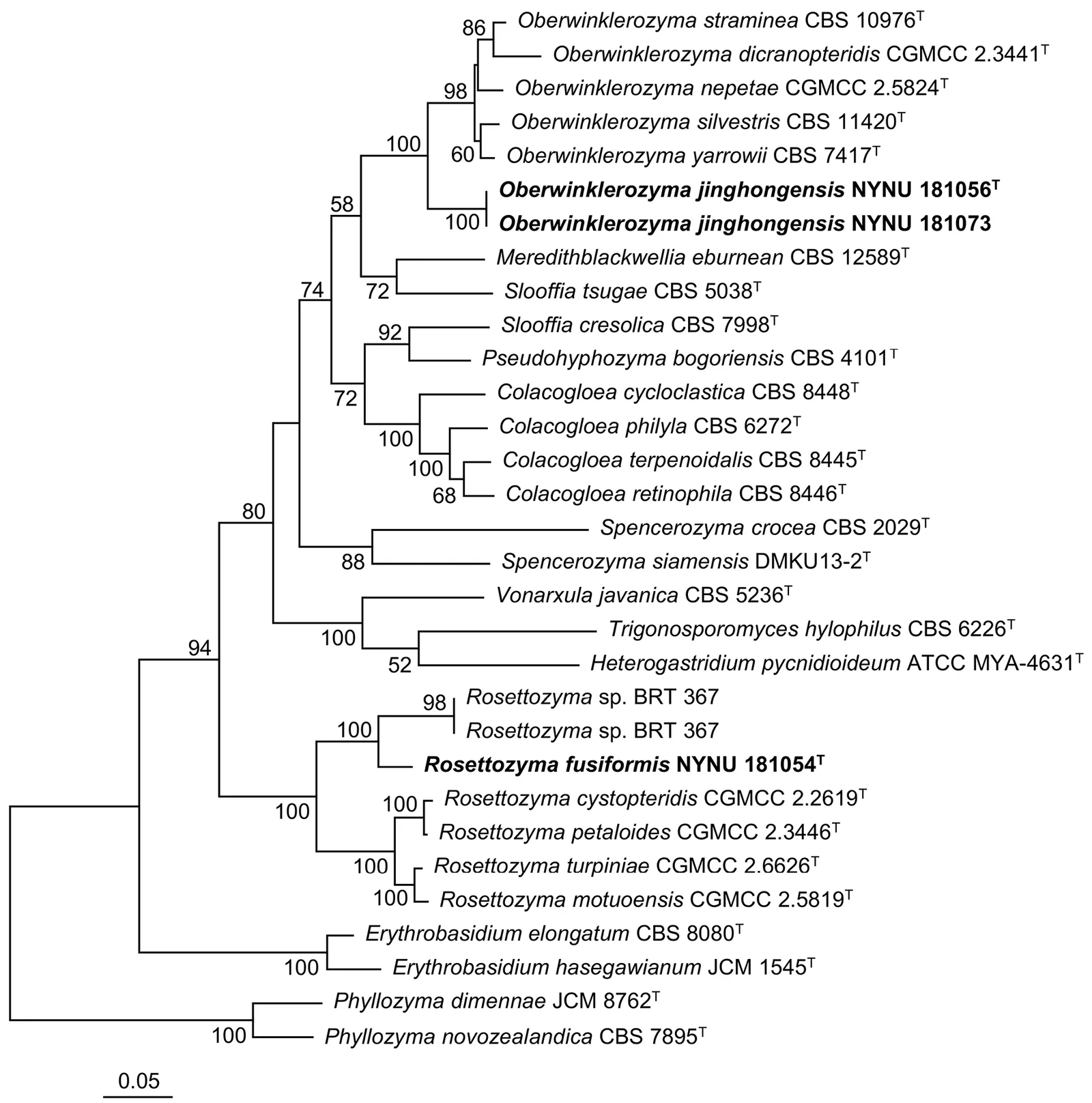
Phylogram of the genera *Oberwinklerozyma* and *Rosettozyma* resulting from a maximum likelihood analysis based on the concatenated D1/D2 and ITS sequences. The tree is rooted with *Phyllozyma dimennae* JCM 8762 and *Phyllozyma novozealandica* CBS 7895. Bootstrap support (BS) ≥50% is shown. Isolates from the present study are marked in bold, and ex-type strains are marked with ^“T”^.

To determine the phylogenetic placement of the two putative novel species, phylogenetic analysis was performed based on a concatenated D1/D2 and ITS dataset. The dataset comprised 31 isolates representing 29 species and 12 genera, with *Phyllozyma dimennae* JCM 8762 and *Phyllozyma novozealandica* CBS 7895 selected as the outgroup taxa. The total length of the concatenated two-locus alignment was 1,166 characters, including 585 bp from the D1/D2 domain and 581 bp from the ITS region. The phylogenetic tree revealed that the three strains represented two distinct novel species belonging to the genera *Oberwinklerozyma* and *Rosettozyma*, respectively (Fig. 1).

Strains NYNU 181056 and NYNU 181073 had identical D1/D2 and ITS sequences, indicating that they are conspecific. These two strains formed a separate basal branch within the *Oberwinklerozyma* clade (Fig. 1). They differed from the other known *Oberwinklerozyma* species by more than 22 nucleotide substitutions (~4.1%) in the D1/D2 domain and 42 nucleotide mismatches (~6.8%) in the ITS region. These molecular differences support the recognition of NYNU 181056 and NYNU 181073 as representing a novel species of *Oberwinklerozyma*, for which the name *Oberwinklerozyma jinghongensis* sp. nov. is proposed.

Strain NYNU 181054 was placed within the *Rosettozyma* clade (Fig. 1). This strain clustered with two unpublished strains, *Rosettozyma* sp. BRT 367 and *Rosettozyma* sp. BRT 395, which were isolated from bromeliads collected in Brazil. Strains BRT 367 and BRT 395 differed from NYNU 181054 by 28 nucleotide substitutions (~5.1%) in the D1/D2 domain, indicating that these unpublished strains may represent different species. Compared with the three known species of *Rosettozyma*, NYNU 181054 differed by 47–50 nucleotide substitutions (~8.2–8.8%) in the D1/D2 domain and 70–75 nucleotide mismatches (~11.6–12.5%) in the ITS region. These levels of sequence divergence support the recognition of NYNU 181054 as representing a novel species of *Rosettozyma*, for which the name *Rosettozyma fusiformis* sp. nov. is proposed.

### Taxonomy

#### Oberwinklerozyma jinghongensis

Taxon classificationFungiMicrobotryalesChrysozymaceae

C.Y. Chai & F.L. Hui sp. nov.

0F6DB40A-592C-5B3A-ABE8-183E3A59F15E

864135

Fig. 2A, B

##### Etymology.

The specific epithet *jinghongensis* refers to the geographic origin of the type strain, Jinghong City, Yunnan.

##### Typus.

China • Yunnan Province, Jinghong City, Xishuangbanna Primeval Forest Park, in rotting wood of an unidentified evergreen tree, August 2016, K.F. Liu, CICC 33363, holotype (preserved in a metabolically inactive state). Ex-type living culture CBS 16008 = NYNU 181056.

##### Description.

On YM agar after 7 days at 25 °C, the streak culture is white cream, butyrous, smooth and glistening with an entire margin. In YM broth after 3 days at 25 °C, cells are ellipsoidal and cylindrical (2.8–4.1 × 5.6–10.3 µm) and occur singly. Budding is polar. After a month in YM broth at 25 °C, a ring and sediment are formed. In Dalmau plate culture on CMA at 25 °C, pseudohyphae are not formed. Sexual structures are not observed on PDA, CMA or V8 agar at 15 and 25 °C. Ballistoconidia are not produced. Glucose fermentation is absent. The following carbon sources are assimilated: glucose, inulin, sucrose, raffinose (weak), melibiose (weak), D-galactose, lactose (weak), trehalose, maltose, melezitose, methyl-α-D-glucoside (weak), cellobiose (weak), salicin (weak), L-sorbose, L-rhamnose (weak), D-xylose (weak), L-arabinose (weak), D-arabinose, d-ribose (weak), ethanol (weak), glycerol, erythritol (weak), ribitol (weak), galactitol (weak), D-mannitol, D-glucitol, *myo*-inositol (weak), DL-lactate, succinate (weak), citrate (weak), d-gluconate, D-glucosamine (weak), 2-keto-D-gluconate, D-glucuronate, and d-glucono-1,5-lactone. Methanol is not assimilated. Nitrate, nitrite, and L-lysine are assimilated as sole nitrogen sources. Ethylamine and cadaverine are not assimilated. Growth is observed at 25 °C, but not at 30 °C. Growth on 50% (w/w) glucose-yeast extract agar is negative. Starch-like substances are not produced. Growth on 50% (w/w) glucose-yeast extract agar is negative. Urease activity and diazonium blue B reaction are positive.

**Figure 2.**
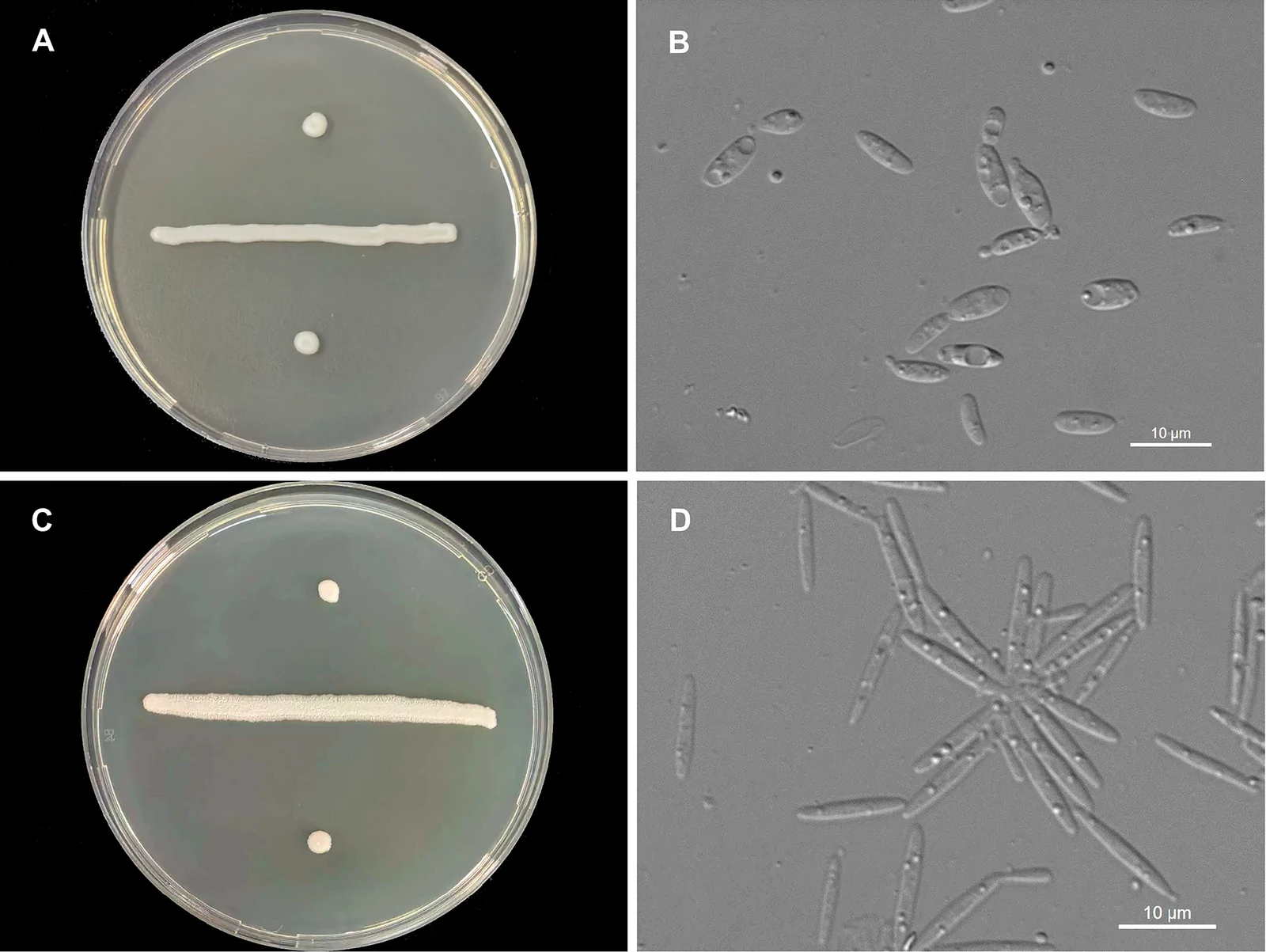
The streak culture grown on YM agar for 7 d at 25 °C and vegetative cells grown in YM broth for 3 d at 25 °C. **A, B**. *O. jinghongensis* NYNU 181056; **C, D**. *R. fusiformis* NYNU 181054.

##### Additional strain examined.

China • Yunnan Province, Jinghong City, Xishuangbanna Primeval Forest Park, in rotting wood, August 2016, K.F. Liu, NYNU 181073.

##### GenBank accession numbers.

Holotype CICC 33363 (D1/D2: MK682806, ITS: MK682798); additional strain NYNU 181073 (D1/D2: MK682806, ITS: MK682798).

##### Note.

Physiologically, *O. jinghongensis* differs from other *Oberwinklerozyma* species in its ability to assimilate inulin, D-ribose, L-rhamnose, and erythritol (Table 2).

**Table 2.** Physiological and biochemical characteristics of the new species and their closely related taxa.

**Characteristic**	**1**	2^*^	3^*^	4^*^	5^*^	6^*^	**7**	8^*^	9^*^	10^*^	11^*^
Assimilation											
Inulin	+	–	–	–	–	–	+	–	–	–	–
D-Ribose	w	–	–	–	–	–	w	v	–	–	–
L-Rhamnose	w	–	–	–	–	–	w	–	–	–	–
Cellobiose	w	+	–	+	+	l	w	v	–	–	–
Lactose	w	+	–	–	–	–	w	v	–	–	–
D-Xylose	w	+	w	–	v	w	–	+	–	–	v
D-Glucosamine	w	+	w	–	+	w	w	–	–	–	–
Glycerol	+	+	l	–	+	l	+	+	–	–	+
Salicin	w	+	–	+	v	l	w	l	+	–	v
Succinate	w	+	+	–	+	+	+	l	–	+	–
Citrate	w	l	l	–	+	l	+	l	–	–	v
Erythritol	w	–	–	–	–	–	w	–	v	–	–
Ribitol	w	+	+	–	v	l	+	v	–	–	–
Galactitol	w	+	–	–	–	–	w	–	–	–	–
Ethylamine	–	+	l	+	n	l	–	l	+	+	+
Growth tests											
Growth at 30 °C	–	–	–	–	–	–	+	–	–	–	–

Note. 1, *O. jinghongensis*; 2, *O. dicranopteridis*; 3, *O. straminea*; 4, *O. nepetae*; 5, *O. yarrowii*; 6, *O. silvestris*; 7, *R. fusiformis*; 8, *R. petaloides*; 9, *R. cystopteridis*; 10, *R. motuoensis*; 11, *R. turpiniae*. +, positive; –, negative; l, latent positive; v, variable; w, weakly positive; n, not available. *Data for reference species were taken from Li et al. (2020) and Sun et al. (2025).

#### Rosettozyma fusiformis

Taxon classificationFungiRosettozymalesRosettozymaceae

C.Y. Chai & F.L. Hui sp. nov.

D829EE2C-6BAC-59E4-9DC9-DD157E6A0DE8

864136

Fig. 2C, D

##### Etymology.

The specific epithet *fusiformis* refers to the fusiform vegetative cells of the type strain.

##### Typus.

China • Yunnan Province, Jinghong City, Xishuangbanna Primeval Forest Park, in rotting wood of an unidentified evergreen tree, August 2016, K.F. Liu, CICC 33362, holotype (preserved in a metabolically inactive state). Ex-type living culture CBS 16007 = NYNU 181054.

##### Description.

On YM agar after 7 days at 25 °C, streak cultures are whitish to cream, butyrous, slightly wrinkled and semi-glossy, and the margin is entire. In YM broth after 3 days at 25 °C, cells are elongate fusiform (1.9–2.1 × 7.6–13.9 µm), either singly or in rosettes. Budding is polar. After a month in YM broth at 25 °C, a sediment and a ring are formed. In Dalmau plate culture on CMA at 25 °C, pseudohyphae are absent. Sexual structures are not observed on PDA, CMA or V8 agar at 15 and 25 °C. Ballistoconidia are not produced. Glucose fermentation is absent. The following carbon sources are assimilated: glucose, inulin, sucrose, raffinose (weak), melibiose (weak), D-galactose, lactose (weak), trehalose, maltose, melezitose, methyl-α-D-glucoside, cellobiose (weak), salicin (weak), L-sorbose, L-rhamnose (weak), L-arabinose (weak), D-arabinose, D-ribose (weak), ethanol (weak), glycerol, erythritol (weak), ribitol, galactitol (weak), D-mannitol, D-glucitol, DL-lactate, succinate, citrate, D-gluconate, D-glucosamine (weak), 2-keto-D-gluconate, D-glucuronate (weak), 5-keto-D-gluconate (weak), and D-glucono-1,5-lactone. D-Xylose, methanol, and *myo*-inositol are not assimilated. Nitrate, nitrite, and L-lysine are assimilated as sole nitrogen sources. Ethylamine and cadaverine are not assimilated. Growth is observed at 30 °C, but not at 35 °C. Growth on 50% (w/w) glucose-yeast extract agar is negative. Urease activity and diazonium blue B reaction are positive.

##### GenBank accession numbers.

Holotype CICC 33362 (D1/D2: MK682808, ITS: MK682801).

##### Note.

Physiologically, *R. fusiformis* differs from other *Rosettozyma* species in its ability to assimilate inulin, L-rhamnose, galactitol, and D-glucosamine and its inability to assimilate ethylamine. Furthermore, *R. fusiformis* is able to grow at 30 °C, whereas other *Rosettozyma* species do not exhibit growth at this temperature (Table 2).

## Discussion

Yeasts are widely distributed and occur in diverse habitats. Rotting wood contains a rich and diverse array of assimilable carbon compounds, making it an important habitat for yeasts (Zheng et al. 2017). Over the past few decades, many yeast species have been isolated from rotting wood collected from different forests worldwide (Santos et al. 2011; Zheng et al. 2017; Varize et al. 2018; Jia et al. 2020; Souza et al. 2023; Barros et al. 2023, 2024; Guo et al. 2024; Garcia-Acero et al. 2024; Hamaguchi et al. 2024). Most of these species are ascomycetous yeasts, particularly members of the genera *Spathaspora*, *Scheffersomyces*, and *Sugiyamaella*, which include naturally xylose-fermenting yeasts and have often been investigated for biotechnological and ecological purposes (Sena et al. 2017; Cadete et al. 2017; Morais et al. 2020). Basidiomycetous yeasts have also been recovered, belonging to the genera *Cryptococcus*, *Phaeotremella*, *Teunia*, *Trichosporon*, *Vishniacozyma*, and *Vanrija* (Jacobson et al. 2003; Middelhoven 2006; Zheng et al. 2017; Xi et al. 2019; Guo et al. 2024). The frequent recovery of ascomycetes may be partly attributable to the use of selective culture media, such as yeast nitrogen base (YNB)-xylose, YNB-cellulose, or YNB-xylan, which allow only specific groups of microorganisms to grow and may therefore not provide a complete picture of the yeast diversity present in rotting wood. Conversely, the use of non-selective enrichment media may increase the likelihood of detecting basidiomycetous yeasts from this substrate (Zheng et al. 2017). However, medium composition is not the only variable; isolation method and incubation temperature may also play important roles in determining the diversity of isolates obtained.

China has been recognized as a region with high potential for the discovery of novel and previously unrecorded yeast species (Boekhout et al. 2021). To date, a total of 69 yeast species have been isolated from rotting wood in China, 39 of which were originally described from this country (Zheng et al. 2017; Shi et al. 2021; Chai et al. 2023). In this study, 21 yeast species were isolated and molecularly identified from rotting wood collected in Xishuangbanna Primeval Forest Park. These included eight previously known species (*Candida tropicalis*, *Lachancea thermotolerans*, *Saitozyma podzolica*, *Schwanniomyces vanrijiae*, *Scheffersomyces insectosa*, *Scheffersomyces stipitis*, *Sugiyamaella americana*, and *Wickerhamiella spandovensis*), 11 newly recorded species (*Apiotrichum laibachii*, *Cutaneotrichosporon cutaneum*, *Hemisphaericaspora subhashii*, *Kurtzmaniella natalensis*, *Ogataea siamensis*, *Petasospora bimundalis*, *Saturnispora diversa*, *Sugiyamaella valdiviana*, *Torulaspora delbrueckii*, *Wickerhamomyces pijperi*, and *Yamadazyma akitaensis*), and two novel species (*O. jinghongensis* and *R. fusiformis*). Among the isolates, *Saitozyma podzolica*—a species previously reported to occur frequently in soils beneath logs—was also recovered, though it was absent from adjacent soils (Yurkov et al. 2012). Although recent studies have advanced the understanding of yeast diversity in rotting wood, the full species richness of this habitat in China remains largely unexplored. This knowledge gap is particularly striking in eastern China, where extensive forest resources exist, but almost no yeast records have been reported from rotting wood. These underexplored regions therefore represent high-priority targets for comprehensive yeast diversity surveys. Such efforts are needed not only to elucidate the ecological roles of these yeasts but also to assess their industrial potential—which may eventually translate into valuable resources for agricultural, environmental, and biotechnological applications.

## Supplementary Material

XML Treatment for Oberwinklerozyma jinghongensis

XML Treatment for Rosettozyma fusiformis

## Additional information

### Artificial Intelligence (AI) use

The authors accept full responsibility for the content of the manuscript, including the disclosure of any use of AI.

No AI tools were used in the preparation of this manuscript.

### Author ORCIDs

L.Tian https://orcid.org/0009-0000-5260-9107

C.-Y. Chai https://orcid.org/0000-0001-7753-6223

Q.-H. Niu https://orcid.org/0000-0003-1695-7117

F.-L. Hui https://orcid.org/0000-0001-7928-3055

### Data availability

All of the data that support the findings of this study are available in the main text.
